# Provincial inequalities in child nutritional risk: a public health multivariate approach using Z-score matrices for spatial vulnerability assessment

**DOI:** 10.3389/fpubh.2026.1766038

**Published:** 2026-04-15

**Authors:** Angel Antonio Morán-Herrera, Melissa Mercedes Idrovo-Hurel, Verónica Patricia Sandoval-Tamayo, Jeylyn Nicole Reyes-Durango, Dennis Alfredo Peralta-Gamboa

**Affiliations:** State University of Milagro, Milagro, Ecuador

**Keywords:** child malnutrition, environmental determinants, public health, social determinants, territorial gaps

## Abstract

**Introduction:**

Child malnutrition remains a persistent public health challenge in Ecuador, characterized by significant territorial inequalities that disproportionately affect children under 5 years of age. These disparities are closely linked to social, environmental, and structural determinants, requiring analytical approaches that go beyond national averages to capture spatial heterogeneity.

**Methods:**

This study employed a quantitative, ecological, and cross-sectional design using secondary data from the National Survey on Child Undernutrition (ENDI 2022–2023). A provincial-level Z-score standardized matrix was constructed to ensure comparability across heterogeneous indicators. Multivariate techniques were applied, including Principal Component Analysis (PCA), HJ-Biplot representation, hierarchical clustering (Ward.D2), and k-means classification, to identify territorial patterns of nutritional vulnerability.

**Results:**

The first two principal components explained 58.47% of total variance, representing structural vulnerability and socio-environmental inequality. Cluster analysis identified three distinct territorial groups (high, medium, and low risk), confirming non-random spatial patterns. Provinces with higher malnutrition prevalence were consistently associated with limited access to drinking water, inadequate sanitation, precarious housing conditions, and lower caregiver education levels. Multivariate analyses revealed strong correlations among environmental and social determinants, highlighting the multidimensional nature of child malnutrition.

**Discussion:**

Findings suggest that child malnutrition in Ecuador is a multi-causal and territorially conditioned phenomenon shaped by persistent structural inequalities. The integration of Z-score standardization with multivariate techniques provides a robust and replicable framework for identifying priority areas and supporting targeted, evidence-based public health interventions.

## Introduction

Child malnutrition remains one of the most urgent and persistent public health challenges in Ecuador. Despite national strategies aimed at improving maternal and child health, strong territorial disparities continue to affect cognitive development, growth, and overall survival in children under 5 years of age. Epidemiological evidence shows that chronic malnutrition is not merely a biological condition; rather, it is rooted in structural inequalities that limit access to quality basic services and are shaped by social, environmental, domestic, and caregiver-related factors. Studies conducted in rural areas and indigenous communities of the Sierra and Amazon regions confirm this multi-causal complexity, demonstrating higher prevalence rates of stunting and anemia where housing, sanitation, education, and food systems are simultaneously inadequate (1–3). These findings underscore the need to address child malnutrition from a territorial and ecosystemic perspective.

According to the National Survey on Child Undernutrition (ENDI 2022–2023) conducted by the National Institute of Statistics and Census (INEC), chronic malnutrition shows an average provincial prevalence of 23.7% across Ecuador, confirming that nearly one in four children under five is affected (4). These figures confirm that child malnutrition remains a structural public health problem rather than a residual condition. Such national evidence underscores the urgency of adopting analytical approaches capable of capturing territorial heterogeneity rather than relying solely on national averages.

Recent research on child health and food insecurity in Ecuador further indicates that rural families face structural constraints that reduce access to nutritious foods, strongly associated with caregivers’ educational levels and unequal access to social protection programs (5). Under these circumstances, multivariate analytical approaches become essential for examining the spatial convergence of these determinants. Territorial analysis provides a lens through which nutritional risks can be understood at the provincial scale, integrating environmental, social, and infrastructural conditions that shape child health outcomes.

However, despite institutional efforts, many interventions remain anchored in a biomedical paradigm that overlooks broader determinants such as environmental health, access to drinking water, housing quality, income, and social context. Traditional studies often evaluate nutritional and social indicators in isolation, which limits the capacity to identify comprehensive territorial patterns needed for effective public health decision-making. Furthermore, Ecuadorian literature still lacks provincial-scale spatial vulnerability assessments that integrate socio-environmental and nutritional indicators using Z-score standardized matrices, which limits systematic territorial comparison across provinces. In contrast, multivariate methods such as cluster analysis, principal component analysis, HJ-Biplot, and the use of Z-score matrices enable the simultaneous examination of numerous variables, revealing underlying structures and territorial heterogeneity. In this context, Z-score standardization is crucial because it allows the comparison of heterogeneous indicators such as access to drinking water, sanitation coverage, and anemia or malnutrition rates on a common scale, reducing bias derived from different units of measurement and enabling more robust provincial comparisons. Heat maps, for example, allow high-dimensional patterns to be visualized (6), while principal component analysis builds on Pearson’s geometric principles (7) and Hotelling’s statistical formalization (8). The HJ-Biplot, combined with classification techniques, is particularly effective for displaying territories and variables within the same factorial space, facilitating the identification of provincial groups sharing similar multivariate nutritional risk profiles.

Therefore, this study aims to analyze provincial inequalities in child malnutrition in Ecuador by integrating nutritional, environmental, and social indicators from the country’s 24 provinces through Z-score standardization and multivariate techniques (PCA, HJ-Biplot, clustering, and heatmaps) to identify territorial risk profiles and the structural factors associated with childhood malnutrition.

## Methodology

This study follows a quantitative, ecological, and cross-sectional design aimed at examining territorial inequalities in child malnutrition across Ecuadorian provinces. The province was defined as the unit of analysis, allowing the identification of spatial patterns and structural vulnerabilities at the territorial level. The observational nature of the study is consistent with ecological approaches commonly used in public health research to explore territorial health disparities.

The methodological approach integrates public health and multivariate statistical perspectives to analyze how social, environmental, and nutritional factors interact territorially. The study relies on secondary data derived from the National Survey on Child Undernutrition (ENDI 2022–2023).

To ensure analytical coherence and reproducibility, the research was organized into a sequential five-phase framework:

1. Data acquisition from official ENDI tabulations,
2. Data cleaning and harmonization,
3. Construction of provincial analytical matrices (original and Z-score standardized),
4. Multivariate statistical analysis using PCA, HJ-Biplot (9), and clustering techniques (10, 11), and
5. Territorial interpretation and classification of nutritional risk profiles.

Data cleaning included verification of internal consistency across provincial tabulations, identification of incomplete or inconsistent records, and harmonization of categorical variables into comparable formats. Indicators presenting excessive missingness, unstable estimates, or lack of comparability across provinces were excluded from multivariate integration. When necessary, categorical responses were recoded into binary or ordinal formats to ensure analytical coherence. These procedures followed standard practices for secondary survey data analysis in public health research and aimed to minimize measurement bias while preserving territorial comparability.

This structured design ensures transparency and methodological rigor when identifying territorial vulnerability patterns associated with child malnutrition.

### Sources of information and variables

We obtained official indicators from the National Survey on Child Undernutrition tabulations. (ENDI 2022–2023), a nationally representative survey conducted by Ecuadorian public institutions using standardized and validated instruments for nutritional and socio-demographic assessment. The ENDI applies internationally recognized methodologies for measuring child nutritional status based on WHO growth standards.

We organized the variables into three analytical dimensions to reflect the multidimensional nature of child malnutrition (12):

#### Nutritional indicators

These included chronic, acute, and global malnutrition indicators derived from anthropometric measurements (height-for-age, weight-for-height, and weight-for-age Z-scores) following WHO classification criteria. These indicators are widely used in epidemiological and public health research to assess child undernutrition.

Child nutritional indicators were defined according to World Health Organization (WHO) growth standards. Stunting (chronic malnutrition) was defined as height-for-age Z-score below −2 standard deviations; wasting (acute malnutrition) as weight-for-height Z-score below −2 standard deviations; and underweight as weight-for-age Z-score below −2 standard deviations. These standardized definitions ensure comparability with international epidemiological studies.

#### Social and caregiver characteristics

Maternal education was categorized according to the ENDI classification levels (no formal education, primary, secondary, and higher education). Care practices and household characteristics correspond to standardized ENDI modules on child feeding, caregiving environment, and household composition. Family structure variables reflect the composition of the household as reported in the survey and are not normative classifications but descriptive socio-demographic categories.

#### Environmental and housing conditions

Housing quality and environmental variables were derived from ENDI household modules and include construction materials, access to drinking water, sanitation facilities, and basic infrastructure. These variables follow national survey classifications that distinguish between adequate and inadequate conditions based on access and material quality.

1. All variables used in this study originate from standardized ENDI instruments, ensuring consistency and comparability across provinces.

**Figure 1:**
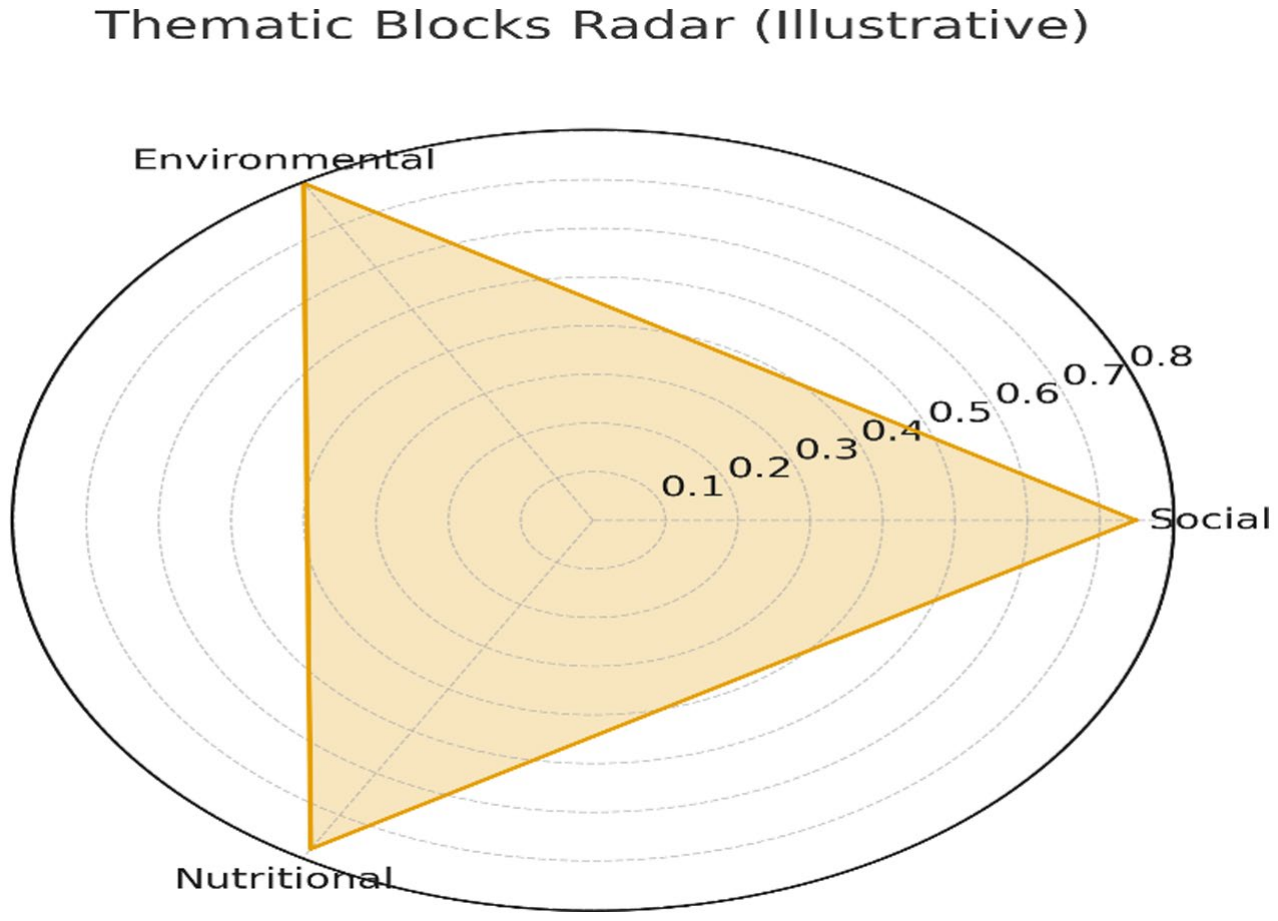
Comparative radar map of the environmental, nutritional, and social thematic segments. Source: Authors’ own elaboration based on ENDI 2022–2023 data.

**Figure 2:**
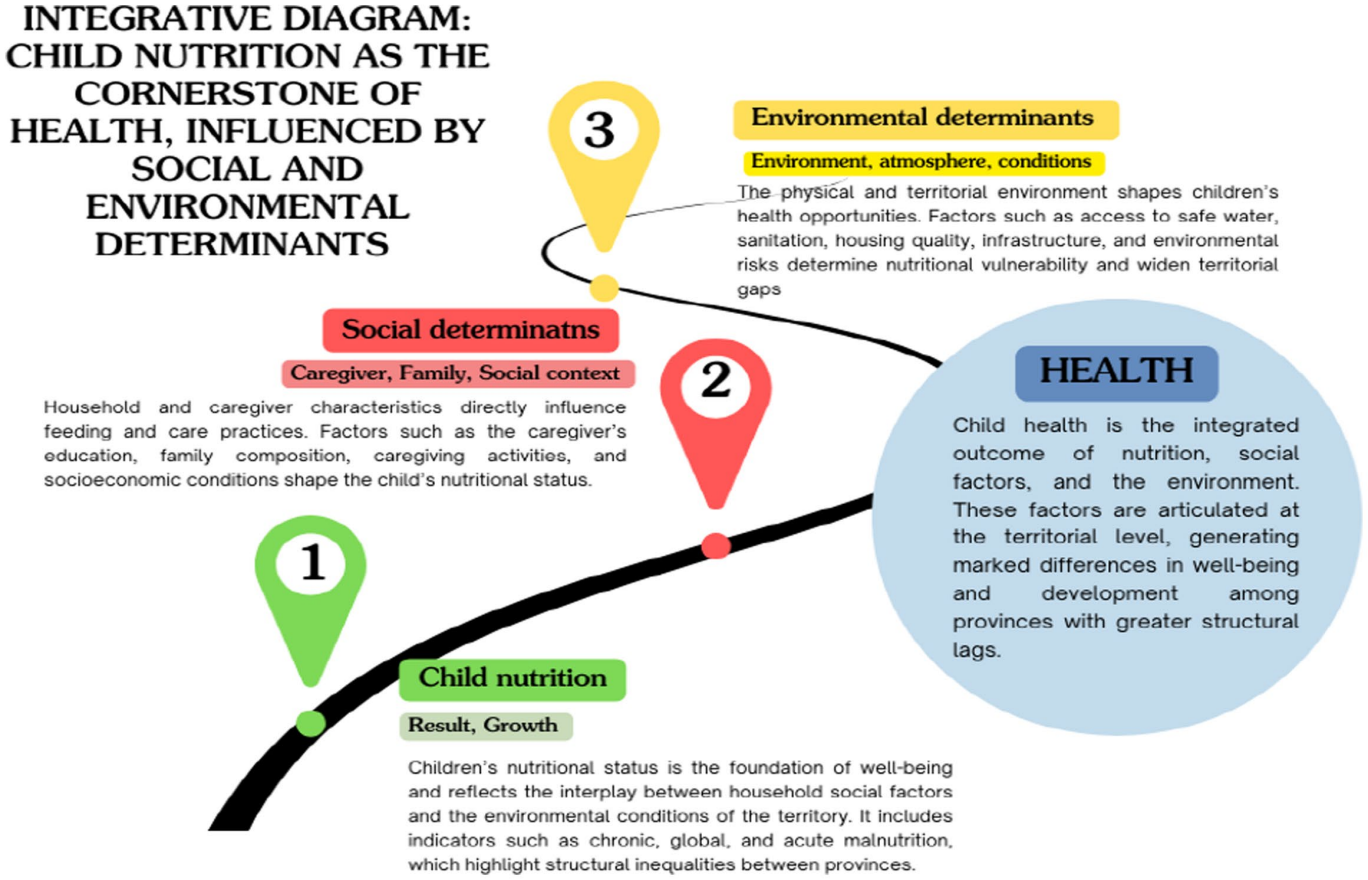
Conceptual interrelation between children’s nutrition, health, and determinants related to social and environmental factors. Source: Authors’ own elaboration based on ENDI 2022–2023 data.

These dimensions were synthesized conceptually in Figure 1 through a comparative radar diagram illustrating the relative weight of each domain. Although not presenting empirical values, the figure visually organizes the analytical axes guiding this research. Figure 2 expands this framework by showing the theoretical interaction between nutritional status and its socio-environmental determinants, supporting the multivariate logic underlying the study. Although ethnic disparities are widely documented in Ecuadorian child nutrition research, the present study could not incorporate ethnic disaggregation due to the provincial aggregation level of the dataset. Future research incorporating ethnicity at the household or individual level would provide a more nuanced understanding of vulnerability patterns.

Based on these indicators, we constructed two provincial-level matrices:

- An original data matrix, and
- A standardized Z-score matrix to ensure comparability across variables with different scales.

The overall analytical structure integrates nutritional, environmental, and social variables to build territorial vulnerability profiles for child malnutrition.

### Final multivariate matrix variables

The final analytical matrix included a set of standardized variables grouped into three dimensions:

- Nutritional indicators: chronic malnutrition (stunting), acute malnutrition (wasting), and underweight prevalence.
- Social and caregiver variables: maternal education level, household composition, and caregiver-related characteristics.
- Environmental and housing variables: access to drinking water, sanitation facilities, housing materials, and basic infrastructure conditions.

All variables were standardized using Z-scores prior to multivariate analysis to ensure comparability across different measurement scales.

### Analytical procedure

**Figure 3:**
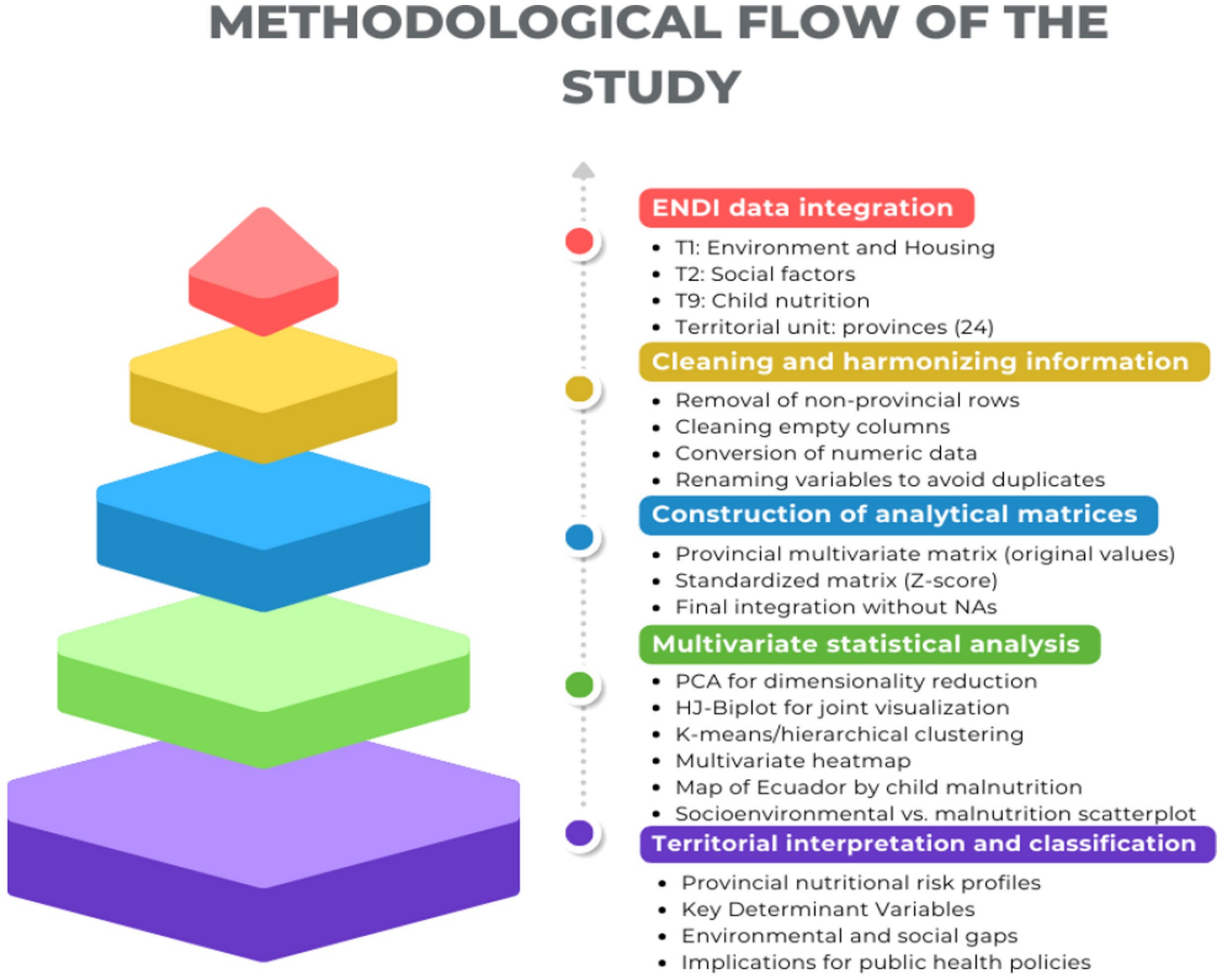
Phases of the analytical method used to investigate nutritional vulnerability at the territorial level. Source: Authors’ own elaboration based on ENDI 2022–2023 data.

Figure 3 illustrates the five-phase analytical framework adopted in this study, summarizing the progression from data acquisition to territorial interpretation. This framework was designed to ensure methodological transparency and reproducibility, showing how data preparation, standardization, and multivariate analyses were sequentially integrated. Rather than representing isolated steps, the phases operate as a coherent analytical flow in which each stage informs the next, enabling robust identification of territorial vulnerability patterns.

### Data processing and software

The first two principal components explained 58.47% of the total variance, with PC1 accounting for 38.76% and PC2 for 19.71%. This proportion of explained variance supports the use of a two-dimensional factorial representation, capturing the main axes of territorial variability associated with child nutritional vulnerability. The retained components reflect the underlying multivariate structure of socio-environmental and nutritional inequalities, enabling a parsimonious and interpretable representation of complex territorial patterns.

This level of explained variance is considered adequate for dimensionality reduction in complex multivariate ecological datasets.

We conducted all multivariate statistical analyses and visualizations using the R programming language (R Foundation for Statistical Computing, R version 4.3.3, packages: fmsb, sf, ggplot2, pheatmap, RColorBrewer, ggrepel, dplyr) within the RStudio integrated development environment. This computational framework ensures transparency, reproducibility, and technical rigor in the implementation of multivariate methods.

Principal Component Analysis (PCA) was applied to reduce dimensionality and identify the main axes of territorial variability from a nutritional perspective. PCA follows its original geometric formulation and later statistical formalization. The use of PCA in studies of socio-environmental determinants has gained relevance in child malnutrition research, particularly when integrated with hierarchical and territorial approaches.

To ensure statistical robustness, the proportion of variance explained by each principal component was evaluated. The first two retained components satisfied the Kaiser criterion (eigenvalues >1) and together accounted for 58.47% of the cumulative variance. This level of explained variance supports the adopted dimensionality reduction strategy and justifies the use of a two-dimensional factorial space to represent the main axes of territorial variability associated with child nutritional vulnerability. The retained components capture the underlying multivariate structure of socio-environmental and nutritional inequalities, enabling a parsimonious and interpretable representation of complex territorial patterns.

The adequacy of the data for multivariate analysis was evaluated using the Kaiser–Meyer–Olkin (KMO) measure and Bartlett’s test of sphericity. The KMO index indicated satisfactory sampling adequacy, while Bartlett’s test was statistically significant (*p* < 0.05), confirming that the correlation matrix was suitable for factor extraction and that the variables exhibited sufficient interdependence for principal component analysis (13).

Recent literature also highlights that food choice and dietary environments are dynamic processes shaped by social and contextual factors, reinforcing the importance of incorporating caregiver and household-level variables in multivariate nutritional analyses (14). Additionally, evidence from Latin America confirms that child stunting remains a major public health concern linked to structural and territorial inequalities, underscoring the need for analytical frameworks capable of capturing multilevel determinants (15).

The HJ-Biplot simultaneously represents provinces and variables within a single factorial space, allowing the identification of factors with the greatest territorial impact. This study adopts the standard formulation.

To complement factorial representation, the analysis applied hierarchical cluster analysis and k-means clustering to classify provinces. The integration of biplot representation with clustering follows a previously proposed biplot-based clustering approach, using the k-means algorithm. This sequential application strengthens the identification of consistent multivariate territorial patterns.

The number of clusters was determined using a combination of statistical and interpretative criteria. Specifically, the dendrogram structure obtained from hierarchical clustering (Ward.D2 method), the elbow method, and the interpretability of cluster separation in the PCA factorial space were jointly considered. The selection of three clusters provided the most parsimonious and statistically consistent classification, balancing intra-group homogeneity and inter-group heterogeneity.

### Ethical considerations

The study did not require ethics committee approval because it used anonymized public data. Principles of scientific integrity, confidentiality, and reproducibility were upheld throughout the research process.

## Results

### Geographic distribution of child malnutrition in Ecuador

Our spatial analysis shows that child malnutrition varies widely across the country’s 24 provinces. We constructed the provincial epidemiological map using the Indicator_T9_i1 indicator, showed a marked concentration of high values in the provinces of the central Sierra and the Amazon. This is related to inadequate environmental conditions, inequality in basic infrastructure, and difficulties in accessing health services.

The variable Indicator_T9_i1 corresponds to the provincial prevalence of chronic child malnutrition as reported in the ENDI 2022–2023 dataset. It is expressed as the percentage of children under 5 years of age presenting height-for-age Z-scores below −2 standard deviations, following WHO classification criteria.

In this study, “inadequate environmental conditions” refer specifically to limited access to safe drinking water, deficient sanitation systems, and precarious housing materials, as defined by ENDI household indicators. For example, several provinces in these regions show below-national-average coverage of piped drinking water and higher proportions of households using unimproved sanitation facilities.

The study categorized provinces with a high nutritional burden as “high” and “very high” (orange-red). In contrast, other provinces, such as Azuay and Pichincha, had lower values. This spatial pattern implies the coexistence of environmental, social, and structural components that affect child nutrition differently. Levels classified as “high” or “very high” correspond to provinces with Z-score values above +1 and +2 standard deviations from the national mean, respectively.

**Figure 4:**
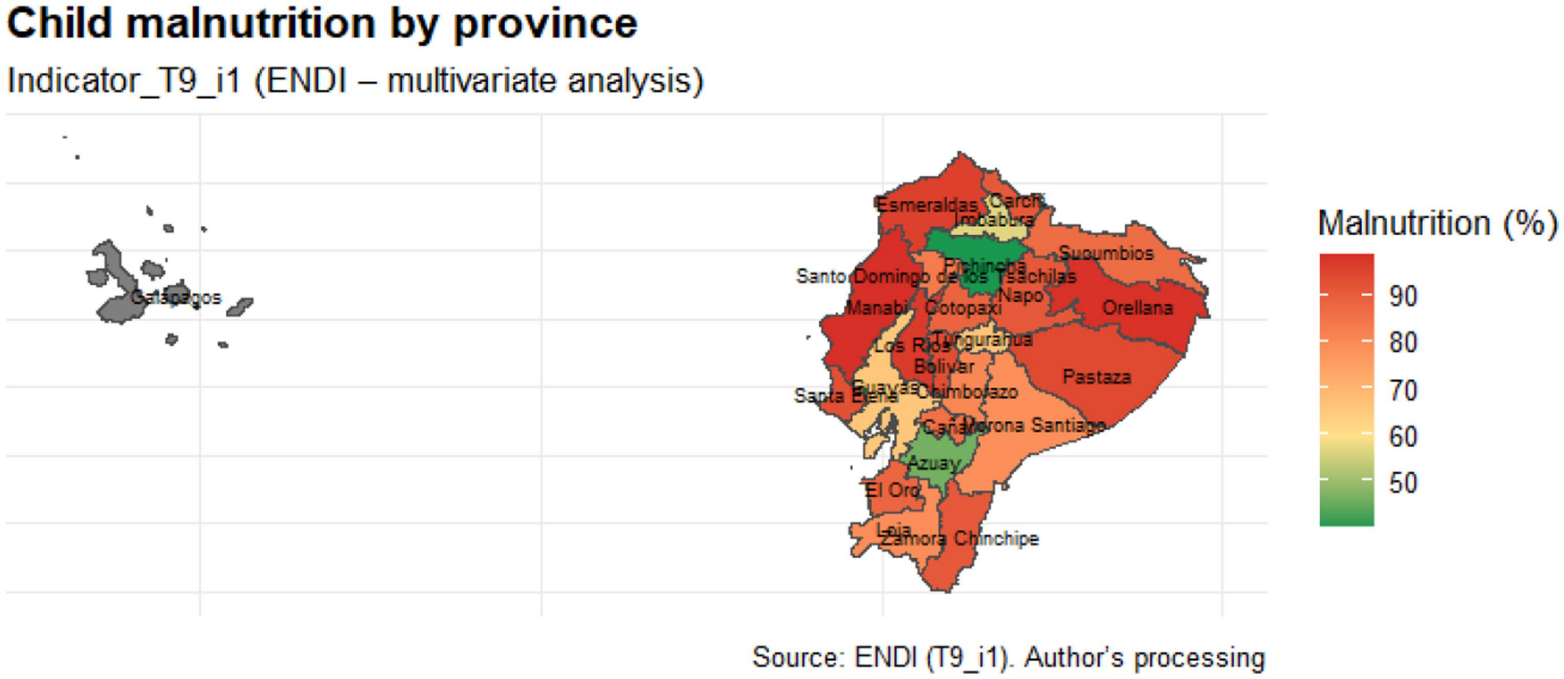
Provincial map of malnutrition in Ecuadorian children. Source: Authors’ own elaboration based on ENDI 2022–2023 data.

The distribution of malnutrition among children across Ecuador’s twenty-four mainland provinces is illustrated in Figure 4. The provinces of the Amazon and the north-central Sierra, which face greater socioeconomic constraints and less access to basic services, as well as geographic barriers that undermine child well-being, exhibit a high prevalence. In contrast, provinces such as Pichincha and Azuay have much lower figures, indicating more extensive access to basic services and superior infrastructure conditions. The 2022–2023 ENDI does not provide statistically significant provincial estimates for this indicator, so the province of Galápagos is shown in gray. Therefore, it was not included in either the comparative interpretation or the statistical analyzes. This map demonstrates the uneven territorial distribution of child malnutrition, rather, it reflects historical territorial disparities that disproportionately affect indigenous, rural, and Amazonian populations.

### Multivariate patterns: interaction of nutritional, environmental, and social factors

A multivariable heatmap, created using the complete Z-score matrix, revealed similarities among the provinces and the six variables.

The dendrograms showed:

- A set of provinces with high nutritional risk, exhibiting deficiencies in drinking water, sanitation, housing materials, caregiver education, and nutritional burden.
- A second set with medium risk, in which some social determinants have higher values, although there are still gaps in the environmental area.
- A third group that benefits, with more positive structural indicators and a lower malnutrition rate.

The heatmap revealed blocks with very high correlation in terms of variables:

1. Environmental factors (water, housing, sanitation), Environmental elements (water, housing, sanitation),
2. Household social determinants, including caregiver education and family structure variables, formed a highly correlated block within the multivariate structure, and
3. Social components within the household and of the caregiver, and purely nutritional factors, which confirms its multifactorial nature.

**Figure 5:**
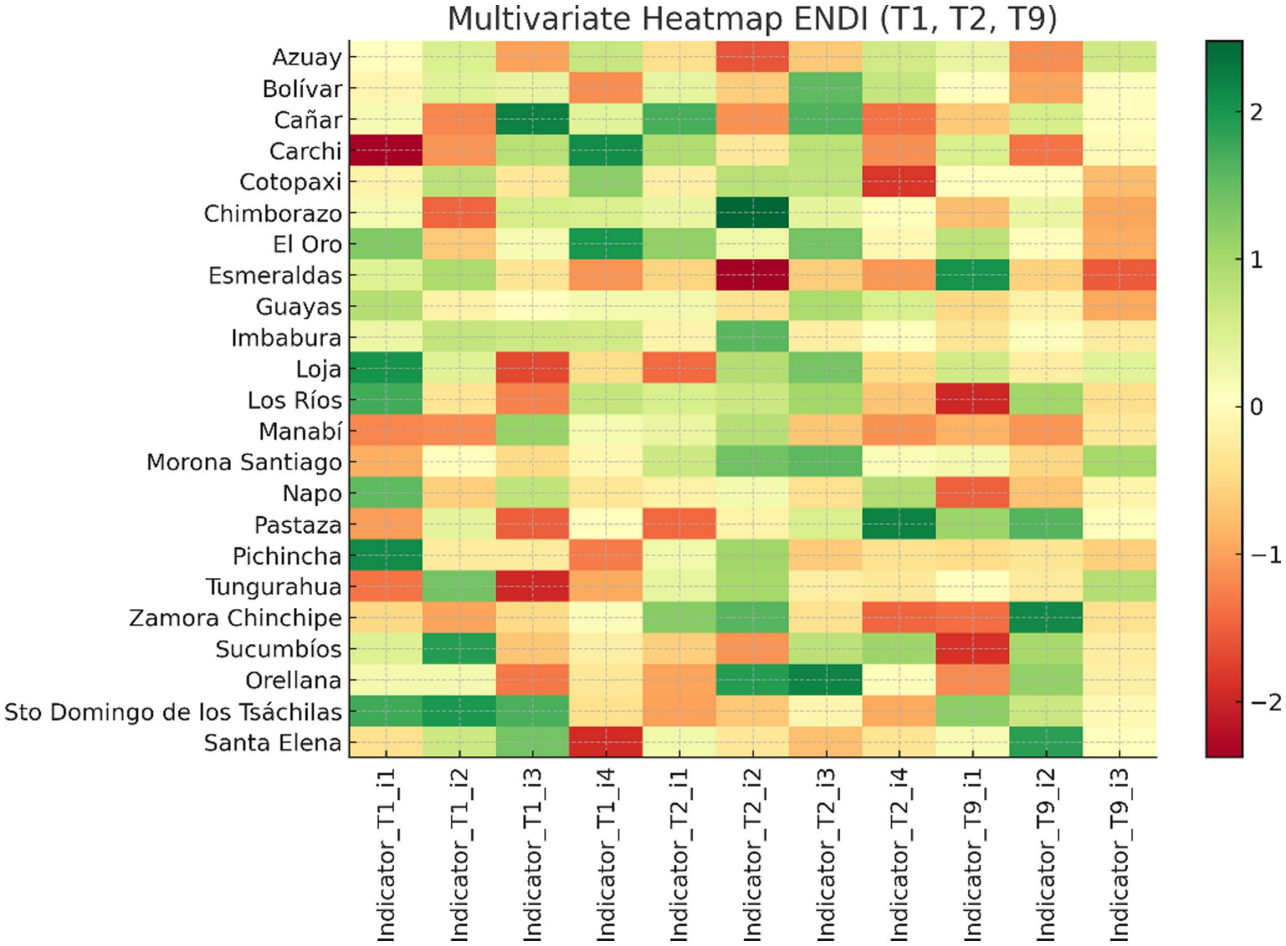
Multivariate heatmap based on the standardized Z-score matrix. Source: Authors’ own elaboration based on ENDI 2022–2023 data.

Figure 5 shows the multivariate heatmap, which was created using the Z-score matrix. This highlights the provinces and main elements that allow for an immediate reading. The provinces of Bolívar., Morona Santiago, Chimborazo, and Napo appear in red across several variables. This indicates that there are vulnerabilities at the social level (caregiver education), nutritional level, and environmental level (water, sanitation, and housing supplies). In contrast, provinces such as Azuay and Pichincha exhibit more nuanced patterns that adapt to more favorable structural conditions. The use of dendrograms and the shading of significant variables makes it possible to identify provincial groups with similar profiles and sets of factors that exhibit a high correlation, confirming that in Ecuador child malnutrition has multiple causes and is geographically concentrated.

### Principal components: latent axes of territorial diversity

The retained components were selected based on the Kaiser criterion (eigenvalues >1). The first two components explained 58.47% of the total variance, supporting the adequacy and stability of the dimensionality reduction. This justifies the use of a two-dimensional factorial space for interpreting territorial patterns of child nutritional vulnerability, as it captures the main axes of multivariate variability underlying socio-environmental inequalities.

PCA retained two components explaining most of the variability:

The first two components explained 58.47% of the total variance, with PC1 accounting for 38.76% and PC2 for 19.71%.

- Component 1 (C1): Structural vulnerability axis, characterized by a high burden of unsafe water, poor hygiene, unsafe materials, and a low level of caregiver education, as well as inadequate economic and social conditions.
- Component 2 (C2): Environmental and sociodemographic axis, which includes various contributions from environmental and social variables.

The connection between child malnutrition and structural precariousness becomes evident because the provinces with the highest C1 scores also had the highest values for Indicator_T9_i1.

**Figure 6:**
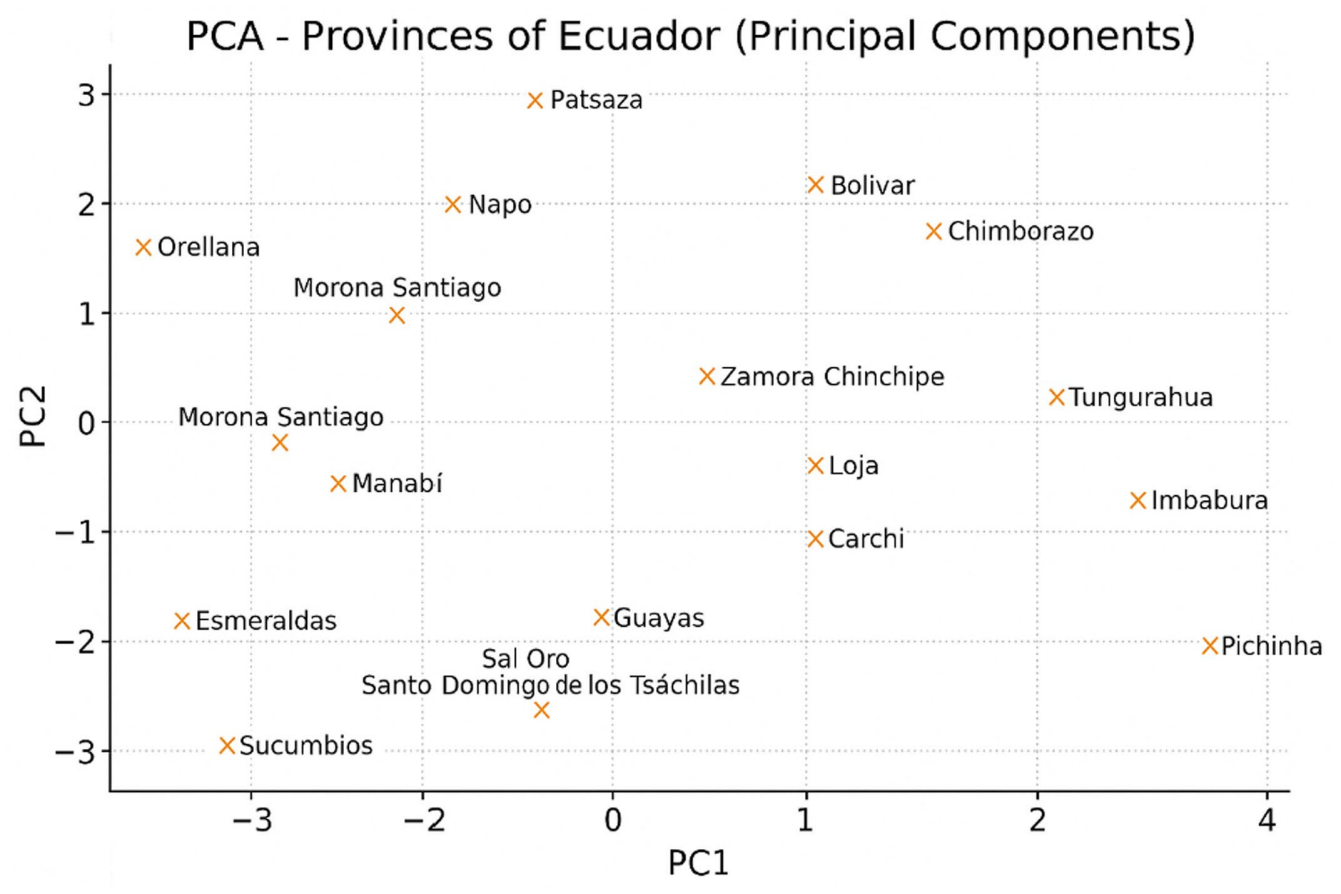
Distribution of Ecuadorian provinces according to the first two principal components (PC1 and PC2). Source: Authors’ own elaboration based on ENDI 2022–2023 data.

The representation of the provinces in the PCA factorial plane is shown in Figure 6. Most of the variation in the dataset is concentrated in the first two components of this plane. The first component synthesizes a socio-environmental hazard linked to education, sanitation, and precarious housing; while the second reveals territorial inequalities associated with environmental and demographic characteristics. The territories with higher malnutrition are located in the region with high values on the first component, confirming that the magnitude of the nutritional problem is determined by the concentration of structural disadvantages. This simplification reveals the underlying structure that organizes territorial risks.

This pattern aligns with national evidence showing that although conditional cash transfers have reduced poverty-related infant mortality, structural territorial disparities remain persistent.

### HJ-Biplot: two-dimensional connections between provinces and key factors

The HJ-Biplot reveals how provinces and determinants interact within the same factorial space. The vectors related to housing, sanitation, and caregiver education variables indicated the provinces where malnutrition is more common. These results suggest that social and environmental deficiencies are closely associated with childhood malnutrition patterns at the territorial level:

- Social and environmental deficiencies are directly related to childhood malnutrition.
- An inverse relationship is established between the availability of essential services and the nutritional indicator.
- Provinces with extreme characteristics (such as Morona Santiago, Bolívar., and Chimborazo) in terms of the tendency for critical vectors to make a higher contribution.

**Figure 7:**
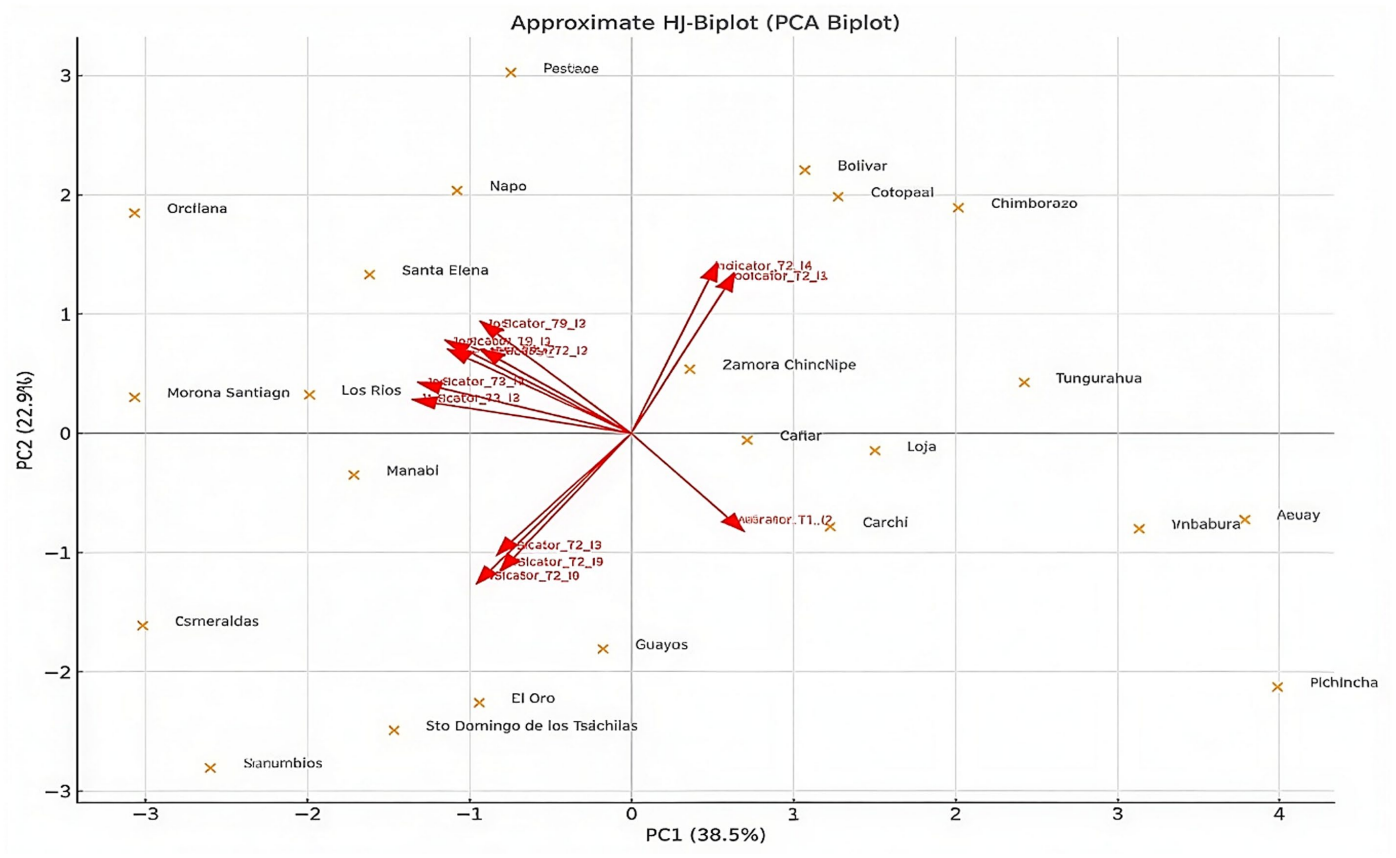
HJ-Biplot of Ecuadorian provinces, based on principal components (PC1–PC2). Source: Authors’ own elaboration based on ENDI 2022–2023 data.

Figure 7 shows the HJ-Biplot plotting provinces in a factorial space along with the directions of the essential factors that determine social, environmental, and nutritional aspects. The relationship between the vectors for housing, sanitation, and caregiver education and the provinces where child malnutrition is most common demonstrates the importance of these factors in determining nutritional risk. The provinces that are most vulnerable tend to align with the direction of these vectors, while the provinces that are better positioned are located at the opposite end of the plane. This graph simultaneously shows the orientation of each factor, its magnitude, and its relationship to the territory.

Beyond its descriptive value, the HJ-Biplot provides an operational framework for territorial decision-making. By simultaneously positioning provinces and structural determinants within the same factorial space, it identifies clusters of accumulated disadvantage that warrant prioritized intervention. This analytical integration strengthens the translation of statistical findings into evidence-based public health policy. By revealing where multiple structural vulnerabilities converge, the HJ-Biplot supports territorial prioritization and more targeted interventions. In this sense, its value extends beyond statistical description to inform evidence-based decision-making.

### Provincial grouping: risk categories regarding nutrition

Thru cluster analysis (Ward.D2 method), Cluster analysis grouped the provinces into three main clusters:

1. Cluster 1—High risk: provinces with high social and environmental vulnerability, as well as the highest rates of child malnutrition.
2. Cluster 2—Medium risk: territories with intermediate conditions; although some factors are advantageous, vulnerabilities still persist.
3. Cluster 3—Low risk: provinces with the most favorable housing conditions, easier access to services, and lower rates of malnutrition.

The correspondence between the provincial map, the PCA, the HJ-Biplot, and the heatmap reveals that the groups are not random but are composed of a mix of social and environmental factors.

**Figure 8:**
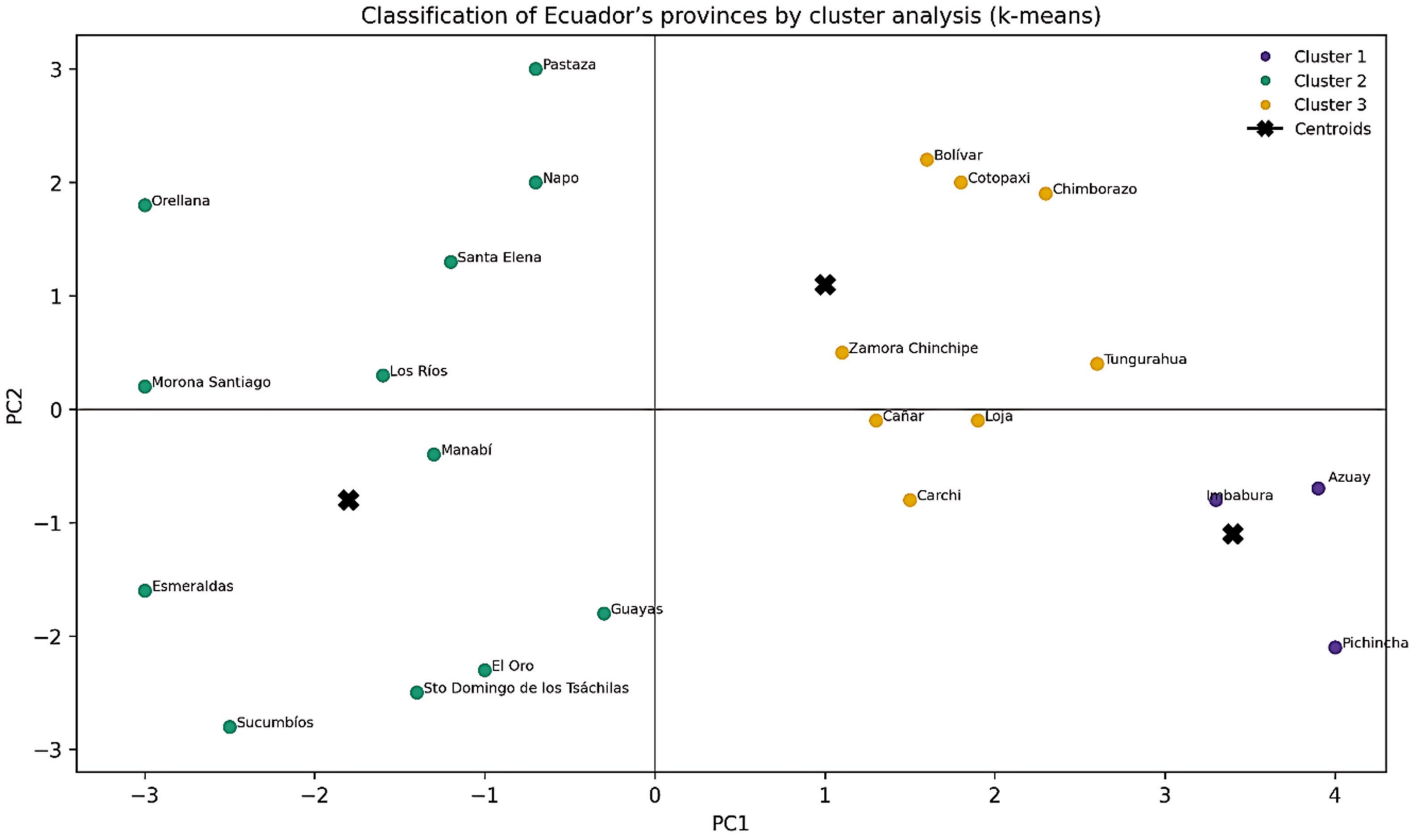
Classification of Ecuador’s provinces by cluster analysis (k-means) based on PC1 and PC2. Source: Authors’ own elaboration based on ENDI 2022–2023 data.

Figure 8 shows the provincial classification obtained with the K-means algorithm, using the two-dimensional PCA projection and the VIRIDIS scientific palette. Each of the three clusters identified is represented by a color, and the “X” s indicate the centroids that best represent the multivariate mean of each cluster. A group of provinces is identified at the extreme of the first principal component, which exhibit greater vulnerability both in terms of nutrition and socio-environmental factors. There is another group with more varied profiles and intermediate vulnerability, and a third group that includes those provinces with lower child malnutrition and better structural conditions. The clear centroids and the distance between the groups demonstrate that there are spatial patterns that coincide with the PCA findings and the heatmap. This supports the multidimensional and geographically determined nature of nutritional risk in Ecuador.

## Discussion

As these data show, child malnutrition in Ecuador exhibits an uneven geographic pattern that is strongly influenced by environmental, social, and structural factors. These territorial disparities coincide with data that have revealed similar patterns of child anemia, chronic malnutrition, and socio-environmental vulnerability in the indigenous and rural populations of the nation (16). Child malnutrition in Ecuador is driven by the cumulative interaction of social, environmental, and infrastructural disadvantages rather than by isolated factors (17).

Above all, in the central highlands and the Amazon, the critical points were located on the provincial map. Historically, these areas have been marginalized and denied access to basic services, healthcare, and economic and social opportunities. These territorial differences are consistent with local research indicating that in areas with limited access to safe drinking water, sanitation, and primary care, child malnutrition increases. These findings reinforce the relevance of a territorial approach to public health planning. Nutritional inequality in Latin America has been associated with socioeconomic status, educational level, and ethnicity (18). Historical national surveys consistently document a substantial ethnic gap in child nutrition. For example, previous Ecuadorian evidence has reported stunting prevalence among Indigenous children exceeding 35%, compared to values often below 20% among mestizo children, reflecting a difference of more than 15 percentage points. This persistent disparity highlights how ethnic inequality intersects with territorial, socioeconomic, and environmental disadvantages, reinforcing the need for targeted and culturally sensitive public health strategies (19, 20).

Given the ecological design of this study, these findings apply to territorial patterns rather than to individual or household-level outcomes. Provincial averages may conceal important intra-provincial heterogeneity, particularly in socially and geographically diverse regions such as the Sierra and Amazon. Therefore, the analysis reveals clear territorial patterns of vulnerability rather than individual nutritional outcomes. Acknowledging this limitation prevents ecological fallacy in policy interpretation.

Beyond these contextual interpretations, this study contributes to the literature by integrating territorial Z-score standardization with multivariate spatial analysis. The observed prevalence levels are also consistent with previous national surveys that have historically placed Ecuador’s chronic malnutrition rates in the 23–27% range, suggesting persistent structural challenges rather than temporary fluctuations.

When viewed in a regional context, these prevalence levels remain high compared to several Latin American countries that have experienced sustained declines in stunting over the last decade. Regional analyses indicate that improvements in sanitation, maternal education, and social protection have contributed to reductions in chronic malnutrition in multiple middle-income countries. In the Latin American context, the coexistence of undernutrition and overweight has also been documented as a growing policy concern, reinforcing the need for integrated nutritional strategies (21).

From a regional perspective, Ecuador’s chronic malnutrition prevalence remains comparatively high among middle-income Latin American countries. Recent evidence indicates that Peru has achieved sustained reductions in stunting through multisectoral nutrition and social protection programs, while Bolivia has shown moderate progress linked to improvements in maternal education and rural health services. In contrast, the persistence of elevated rates in Ecuador suggests that structural territorial inequalities and uneven access to basic services continue to limit nutritional gains. Situating Ecuador within this regional context reinforces the relevance of territorial and multivariate approaches to better understand and address persistent disparities (22, 23).

The primary contribution of this study lies in its integrated territorial perspective, which combines Z-score standardized matrices with HJ-Biplot visualization and clustering techniques to identify multivariate nutritional vulnerability profiles at the provincial scale. By moving beyond single-indicator approaches, this framework captures the interaction between social, environmental, and nutritional determinants. Moreover, the methodological design offers a replicable model for territorial health assessment in middle-income countries facing persistent structural inequalities (24, 25).

Consistent with the multivariate results, the territorial clustering observed aligns with previous evidence the coexistence of high malnutrition rates with limited caregiver education, deficient housing conditions, and inadequate access to water and sanitation in the most vulnerable provinces (26).

Principal Component Analysis further condenses these relationships into latent dimensions that explain 58.47% of territorial variability. The first component captures a structural vulnerability axis integrating social and environmental deprivation, while the second reflects demographic and spatial heterogeneity that may be associated with urbanization dynamics and unequal service distribution.

The HJ-Biplot complements this interpretation by simultaneously positioning provinces and determinants within the same factorial space, showing that territories with higher malnutrition prevalence align closely with vectors representing sanitation deficits and limited caregiver education. This spatial configuration is consistent with eco-territorial and biopsychosocial models that conceptualize child nutrition as dependent on material living conditions and household social capital (27, 28).

Finally, clustering techniques translate these statistical patterns into operational territorial groupings. The identified clusters highlight differentiated provincial realities in which structural disadvantages accumulate, thereby offering a practical framework for targeted public health prioritization. Such differentiated approaches align with a broad body of evidence. Research has shown that improvements in water and sanitation are central to reducing stunting, while other studies highlight the decisive role of maternal education and healthcare access in shaping child nutritional outcomes. Complementary analyses further stress that social protection systems are critical for sustaining long-term nutritional gains in vulnerable populations (29).

Additionally, children’s health and nutrition are affected by the family environment. Ayala and his collaborators point out that factors such as family stability, social cohesion, and environmental quality influence nutritional risk, especially in situations of structural vulnerability. The contextual interpretation of the identified provincial clusters is confirmed by the results of this study (30, 31).

By combining information from the ENDI with multivariate methods, this study advances and enables a comprehensive understanding of the factors contributing to child malnutrition in Ecuador. What they discover can strengthen community-based social work efforts, help create contextualized programs, and drive environmental and territorial measures that respond to the needs of the most underserved areas. The analytical approach adopted in this study was implemented using the R statistical environment, ensuring transparency, reproducibility, and technical rigor in the multivariate analyses (32, 33).

These findings are consistent with previous research that frames child malnutrition not only as a health issue but also as a territorial development challenge shaped by structural inequalities (34).

## Conclusion

Child malnutrition in Ecuador is not randomly distributed but reflects a structurally embedded and territorially differentiated pattern of vulnerability. The multivariate approach applied in this study demonstrates that nutritional risk is shaped by the convergence of socioeconomic, environmental, and service-access inequalities, which interact across provinces in systematic ways.

The identification of clear territorial clusters confirms that provinces with higher nutritional risk simultaneously exhibit multiple structural deficits, reinforcing the need to move beyond isolated interventions toward integrated and territorially targeted policy responses. These findings highlight the importance of incorporating multivariate and spatially informed analytical frameworks into public health planning.

From a methodological perspective, the combined use of PCA, HJ-Biplot, and cluster analysis proves to be a robust strategy for revealing latent structures in complex social datasets, offering a replicable analytical framework for similar contexts in Latin America and other developing regions.

Ultimately, this study contributes to reframing child malnutrition as a multidimensional and territorially conditioned phenomenon, emphasizing that effective policy responses must address the structural determinants underlying nutritional vulnerability rather than focusing solely on immediate outcomes.

### Limitations of the study

Several limitations should be considered when interpreting these findings. First, the analysis relies on secondary data from the ENDI, which may contain reporting errors, vary by sample, or not be representative in provinces with low population density, and whose quality depends on the sample survey. Although indicators with validated estimates were used and standard errors and coefficients of variation were taken into account, the accuracy of some values may compromise the ability to compare different areas.

Secondly, limitation is the absence of ethnic disaggregation at the provincial aggregation level. Given the well-documented ethnic inequalities in child nutrition in Ecuador, this restriction may underestimate vulnerability patterns affecting indigenous and Afro-descendant populations. Future studies incorporating microdata with ethnic identifiers would allow a more precise equity-focused assessment.

Third, the cross-sectional design of the ENDI survey precludes causal inference. While multivariate techniques reveal structural associations and latent territorial configurations, they do not establish temporal or causal pathways. Longitudinal or panel-based designs would be necessary to assess dynamic changes in vulnerability patterns and policy impact over time.

Furthermore, although fundamental social, environmental, and nutritional components were incorporated, the survey modules limit the availability of certain indicators. The study could not fully integrate variables such as water quality, access to healthcare, household food security, and extreme weather conditions due to a lack of uniform information at the provincial level.

The robustness of the territorial mapping and multivariate analyses depends on the consistency and quality of data standardization procedures. Although cleaning and verification processes have been carried out, undetected inconsistencies may still exist in the original tabulations that could alter the interpretation of the patterns. However, the methods used provide a robust and complementary view to better understand territorial inequalities in child malnutrition.

## Data Availability

Publicly available datasets were analyzed in this study. This data can be found at: https://www.ecuadorencifras.gob.ec/institucional/home/.

## References

[1] Ortiz J Van Camp J Wijaya S Donoso S Huybregts L. Determinants of child malnutrition in rural and urban Ecuadorian highlands. Public Health Nutr. (2014) 17:2122–30. doi: 10.1017/S1368980013002528, 24073991 PMC11108716

[2] Rivadeneira MF Moncayo AL Cóndor JD . High prevalence of chronic malnutrition in indigenous children under 5 years of age in Chimborazo-Ecuador: multicausal analysis of its determinants. BMC Public Health. (2022) 22:1977. doi: 10.1186/s12889-022-14327-x, 36307789 PMC9617340

[3] Instituto Nacional de Estadística y Censos (INEC). Encuesta Nacional sobre Desnutrición Infantil (ENDI) 2022–2023: Resultados Oficiales. Quito, Ecuador: Resultados oficiales. Instituto Nacional de Estadística y Censos (INEC) (2023).

[4] Rivadeneira MF Cadena N. Anemia in early childhood and associated factors in Ecuador: an analysis from a health social determinants model. Food Sci Nutr. (2025) 13:e70805. doi: 10.1002/fsn3.70805, 40837496 PMC12362182

[5] Cordero-Ahiman OV Vanegas JL Beltrán-Romero P Quinde-Lituma ME. Determinants of food insecurity in rural households: the case of the Paute River basin of Azuay Province, Ecuador. Sustainability. (2020) 12:946. doi: 10.3390/su12030946 PMC7923421 33672453

[6] Moncayo AL Granizo G Grijalva MJ Rasella D. Strong effect of Ecuador’s conditional cash transfer program on childhood mortality from poverty-related diseases: a nationwide analysis. BMC Public Health. (2019) 19:1132. doi: 10.1186/s12889-019-7457-y, 31420035 PMC6697994

[7] Wilkinson L Friendly M. The history of the cluster heat map. Am Stat. (2009) 63:179–84. doi: 10.1198/tas.2009.0033

[8] Pearson K. On lines and planes of closest fit to systems of points in space. Philos Mag. (1901) 2:559–72. doi: 10.1080/14786440109462720

[9] Hotelling H. Analysis of a complex of statistical variables into principal components. J Educ Psychol. (1933) 24:417–41. doi: 10.1037/h0071325

[10] Galindo-Villardón MP. Una alternativa de representación simultánea: HJ-Biplot. Questiió. (1986) 10:13–23.

[11] Vicente-Villardón JL Galindo MP Blázquez-Zaballos A. Logistic biplots. Multiple correspondence analysis and related methods. London: Chapman & Hall. (2006) 503–521.

[12] Kaiser HF. The application of electronic computers to factor analysis. Educ Psychol Meas. (1960) 20:141–51. doi: 10.1177/001316446002000116

[13] MacQueen J. "Some methods for classification and analysis of multivariate observations". In: Proceedings of the Fifth Berkeley Symposium on Mathematical Statistics and Probability, Berkeley: University of California Press (1967) p. 1:281–97. Available online at: http://projecteuclid.org/euclid.bsmsp/1200512992

[14] Garza M Abascal Miguel L. Health disparities among indigenous populations in Latin America: a scoping review. Int J Equity Health. (2025) 24:119. doi: 10.1186/s12939-025-02495-2, 40307795 PMC12044809

[15] Downs S Bell W Blake CE. Measuring diets and food choice in the context of a changing world. Front Nutr. (2025) 12:1571706. doi: 10.3389/fnut.2025.1571706, 40568421 PMC12189019

[16] Rojas-Montenegro C Gómez G Hincapié O Dvoretskiy S De Witt T Gracia D . The pediatric global burden of stunting: focus on Latin America. Lifestyle Med. (2022) 3:e67. doi: 10.1002/lim2.67

[17] Sandler A Sun L. The socio-environmental determinants of childhood malnutrition: a spatial and hierarchical analysis. Nutrients. (2024) 16:2014. doi: 10.3390/nu16132014, 38999762 PMC11243526

[18] Batis C Mazariegos M Martorell R Gil A Rivera JA. Malnutrition in all its forms by wealth, education and ethnicity in Latin America: who are more affected? Public Health Nutr. (2020) 23:s1–s12. doi: 10.1017/S136898001900466X, 32900396 PMC10200386

[19] Lee GO Gutierrez C Castro Morillo N Cevallos W Jones AD Eisenberg JN. Multiple burdens of malnutrition and relative remoteness in rural Ecuadorian communities. Public Health Nutr. (2021) 24:4591–602. doi: 10.1017/S1368980020004462, 33155533 PMC10195250

[20] Pinzón-Flórez CE Fernández-Niño JA. Determinants of performance of health systems concerning maternal and child health: a global approach. PLoS One. (2015) 10:e0120747. doi: 10.1371/journal.pone.0120747, 25822246 PMC4378969

[21] Fattore GL Santos CAT Barreto ML. Social determinants of childhood asthma symptoms: an ecological study in urban Latin America. J Community Health. (2014) 39:355–62. doi: 10.1007/s10900-013-9769-7, 24046215

[22] López-Cevallos DF Chi C. Evaluación del contexto de la utilización de la atención médica en Ecuador: Un análisis espacial y multinivel. BMC Health Serv Res. (2010) 10:64. doi: 10.1186/1472-6963-10-64, 20222988 PMC2850335

[23] de Onis M Branca F. Childhood stunting: a global perspective. Matern Child Nutr. (2016) 12:12–26. doi: 10.1111/mcn.12231, 27187907 PMC5084763

[24] Smith LC Haddad L. Reducing child undernutrition: past drivers and priorities for the post-MDG era. World Dev. (2015) 68:180–204. doi: 10.1016/j.worlddev.2014.11.014

[25] Ayala GX Monge-Rojas R King AC Hunter R Berge JM. The social environment and childhood obesity: implications for research and practice in the United States and countries in Latin America. Obes Rev. (2021) 22:e13246. doi: 10.1111/obr.13246, 33951272 PMC8365653

[26] Danaei G Andrews KG Sudfeld CR Fink G McCoy DC Peet E . Risk factors for childhood stunting in 137 developing countries: a comparative risk assessment analysis. PLoS Med. (2016) 13:e1002164. doi: 10.1371/journal.pmed.1002164 27802277 PMC5089547

[27] Béné C Oosterveer P Lamotte L Brouwer ID de Haan S Prager SD . When food systems meet sustainability—current narratives and implications for actions. World Dev. (2019) 113:116–30. doi: 10.1016/j.worlddev.2018.08.011

[28] Cumming O Cairncross S. Can water, sanitation and hygiene help eliminate stunting? Current evidence and policy implications. Matern Child Nutr. (2016) 12:91–105. doi: 10.1111/mcn.12258, 27187910 PMC5084825

[29] Grajeda R Hassell T Ashby-Mitchell K Uauy R Nilson E. Regional overview on the double burden of malnutrition and examples of program and policy responses: Latin America and the Caribbean. Ann Nutr Metab. (2019) 75:139–43. doi: 10.1159/000503674, 31743911

[30] Prendergast AJ Humphrey JH. The stunting syndrome in developing countries. Paediatrics Int. Child Health. (2014) 34:250–65. doi: 10.1179/2046905514Y.0000000158, 25310000 PMC4232245

[31] Larrea C Montalvo P Ricaurte AM. Child Malnutrition, Social Development and Health Services in the Andean Region.Research Department Publications 3189. Washington, DC: Inter-American Development Bank. (2005).

[32] Nandy S Miranda JJ. Overlooking undernutrition? Using a composite index of anthropometric failure to assess how underweight misses and misleads the assessment of undernutrition in young children. Soc Sci Med. (2008) 66:1963–6. doi: 10.1016/j.socscimed.2008.01.021, 18299166 PMC2685640

[33] Allaire JJ. RStudio: Integrated Development Environment for R [Software]. Boston, MA: RStudio, PBC (2011).

[34] Posit Team. RStudio: Integrated Development Environment for R. Boston, MA: Posit Software, PBC (2024). Available online at: http://www.posit.co/

